# 3D Printed Multi-Functional Hydrogel Microneedles Based on High-Precision Digital Light Processing

**DOI:** 10.3390/mi11010017

**Published:** 2019-12-23

**Authors:** Wei Yao, Didi Li, Yuliang Zhao, Zhikun Zhan, Guoqing Jin, Haiyi Liang, Runhuai Yang

**Affiliations:** 1 Department of Biomedical Engineering, Anhui Medical University and Research and Engineering Center of Biomedical Materials, Anhui Medical University, Hefei 230032, China; yaowei971020@163.com (W.Y.); lidi970113@gmail.com (D.L.); 2 School of Control Engineering, Northeastern University at Qinhuangdao, Qinhuangdao 066004, China; zhaoyuliang@neuq.edu.cn; 3 Key Lab of Industrial Computer Control Engineering of Hebei Province, School of Electrical Engineering, Yanshan University, Qinhuangdao 066004, China; zkzhan@ysu.edu.cn; 4 Robotics and Microsystems Center, School of Mechanical and Electric Engineering, Soochow University, Suzhou 215021, China; gqjin@suda.edu.cn; 5 CAS Key Laboratory of Mechanical Behavior and Design of Materials, University of Science and Technology of China, Hefei 230027, China; hyliang@ustc.edu.cn; 6 IAT-Chungu Joint Laboratory for Additive Manufacturing, Anhui Chungu 3D printing Institute of Intelligent Equipment and Industrial Technology, Wuhu 241200, China

**Keywords:** microneedle, drug injection, drug detection, 3D printing

## Abstract

Traditional injection and extraction devices often appear painful and cumbersome for patients. In recent years, polymer microneedles (MNs) have become a novel tool in the field of clinical medicine and health. However, the cost of building MNs into any shapes still remains a challenge. In this paper, we proposed hydrogel microneedles fabricated by high-precision digital light processing (H-P DLP) 3D printing system. Benefits from the sharp protuberance and micro-porous of the hydrogel microneedle, the microneedle performed multifunctional tasks such as drug delivery and detection with minimally invasion. Critical parameters for the fabrication process were analyzed, and the mechanical properties of MNs were measured to find a balance between precision and stiffness. Results shows that the stiffness and precision were significantly influenced by exposure time of each layer, and optimized printing parameters provided a balance between precision and stiffness. Bio-compatible MNs based on our H-P DLP system was able to execute drug injection and drug detection in our experiments. This work provided a low-cost and fast method to build MNs with 3D building, qualified the mechanical performance, drug injection, drug detection ability of MNs, and may be helpful for the potential clinical application.

## 1. Introduction

Traditional injection and extraction devices such as hypodermic needles often appear painful and cumbersome for patients. In recent years, polymer microneedles (MNs) have become a novel tool in the field of clinical medicine and health [1]. With the sharp protuberance of their surface, they can inject or extract with minimally invasion which is painless and convenient. For example, microneedles can be used to inject vaccines [2,3,4], and microneedle sensors can be used to detect potassium concentrations in the body [5]. Besides, glucose [6], alcohol [7], lactic acid [8], and benzoquinone [9] can also be measured. There are many sorts of polymer microneedles, such as solid MNs, coated MNs, dissolving MNs, and Hydrogel-Forming MNs. Solid MNs are utilized in non-coated quadratic process, by which the micropore is firstly generated on skin and then the drug is delivered through the micropore. Thus, this method is relatively rough and could cause irreversible wound [10]. Coated MNs, which coats the surface of polymer microneedles with drug and penetrates directly through the cuticle of the skin. However, its dosage is difficult to control and quantify [11]. Dissolving MNs use biocompatible polymers to encapsulate drugs such as viral inactivated vaccines, which could penetrate skin and dissolve automatically within minutes [12]. Recently, Hydrogel-Forming Microneedles are developed because of its’ high-biocompatibility and natural porous structure. Through the diffusion of water, MNs arrays could be controlled to expand and form in-situ hydrogel conduit [13]. In addition to injecting drugs, fluorescent substances could be mixed into microneedle-forming materials to detect antigen. For example, the multiple specificity detection of skin interstitial fluid (ISF) biomarkers by MNs combined with photonic crystal (PhC) barcodes [14].

Most of these polymer microneedles are typically fabricated by template driven [15]. The shape of microneedles built by this way are standard and the material is convenient to change. However, due to the expensive cost of building a template, it is problematic if any modification should be performed to the MNs [16]. Micro-nano 3D technologies are able to build materials into arbitrary shapes and have been employed to directly manufacture MNs without template. The principles of 3D printing contain fused deposition modeling (FDM), selective laser sintering (SLS), and stereolithography (SLA). The precision of FDM is influenced by various factors, such as temperature and large scale of release nozzle, which is hard to satisfy the printing precision of microneedles [17]. Similar limitations exist in SLA and SLS, while the minimum feature size of SLS and SLA is larger than 100 microns [18]. Moreover, SLS requires high power supply and printing temperature, which would raise the cost of fabrication. Ultraviolet (UV) photosensitive resin is commonly used in SLA, but photosensitive resin has poor biocompatibility [19,20]. At the same time, there are some methods combining template driven fabrication with UV curing materials, which make materials cross-linking into strong structure [21,22]. But the fabrication process is complex. 

In this paper, we brought hydrogel MNs based on 3D printing. A self-built high-precision digital light processing (H-P DLP) system based on light curing was utilized to print MNs. The H-P DLP system has high printing accuracy and is capable of meeting precision of building micro-needle [23]. Besides, the cost of H-P DLP system is low, which greatly reduces the cost of micro-needle manufacturing platform comparing to template driven fabrication. In addition, hydrogel could be printed into micro-needles of specific shapes after modeling and slicing. Once the structure of micro-needles needs to be adjusted, only three-dimensional models of micro-needles need to be modified and sliced, which is convenient and economy. MNs with different exposure time were printed and the mechanical properties of those MNs were studied in detail. Moreover, we designed experiments to certify the ability of drug injection and drug detection by MNs. Qualitative and quantitative results are displayed.

## 2. Materials and Methods 

**Figure 1:**
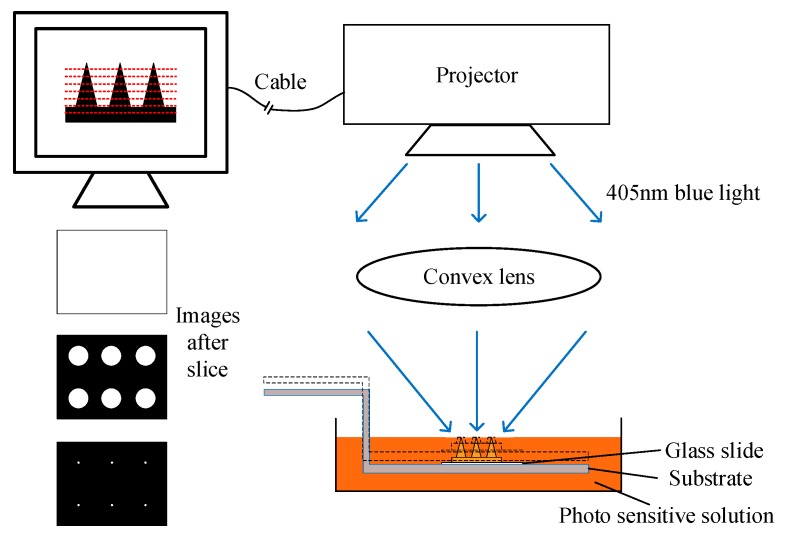
Simplified schematic of high-precision digital light processing (H-P DLP) system.

The raw material of MNs was liquid at first, the monomer in solution would crosslink under the illumination of blue light. The preparation of precursor of MNs was as followed: First, transfer 10 mL Polyethylene glycol diacrylate (PEG400DA, monomer, molecular weight 508, ROJI, Minheng Co., Ltd., Hefei, China) into a brown sealing bottle, then 1% wt Phenylbis (2,4,6-trimethylbenzoyl) phosphine oxide (819, photo-initiator, ROJI) was added into the bottle. Stirring for 24 h. All operations were down under dark environment because of the light sensitivity of photo-initiator. Then we use H-P DLP system made in our previous work to build precursor into solid state MNs [23]. The simplified schematic of the H-P DLP system is shown in Figure 1. The 3D model of MNs was designed in computer and sliced into many cross-sectional images. The interval between neighboring red lines represented the thickness of slices. After slicing, images in different part were sent to projector and displayed one by one. The optical channel of the system is vertical, and the top of the system is a blue light projector which was refitted from a commercial projector. Blue light at 405 nm was illuminated to a convex lens in the middle of platform and was refocused by the convex lens. At last, blue light would be projected onto the surface of the precursor. Precursor would solidify when it was exposed to blue light. The exposure time would influence the precision and stiffness of MNs significantly. Then the substrate would move downward for a distance same as the slice thickness downward so that precursor would cover the surface again. Steps above were repeated until all sliced images were projected one by one and finally, MNs was built over.

Due to the significant impact of exposure time on the precision and stiffness, groups of printing experiments with exposure time of ranging from 50 ms to 900 ms were carried out. MNs with ideal precision and stiffness were used in following drug injection and drug detection experiments.

**Figure 2:**
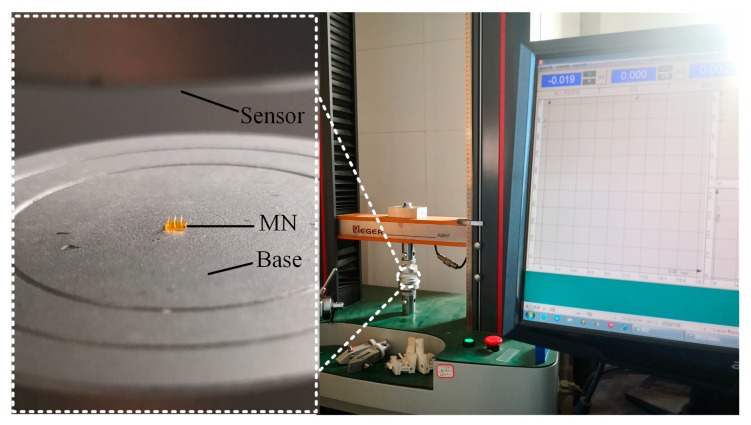
Measuring the mechanical performance of microneedles (MNs) with universal material testing machine.

Universal material testing machine was utilized to measure the mechanical properties of MNs (see Video S1). As shown in Figure 2, the right is the overview picture of testing machine. The left side is the enlarged picture of the white dotted circle in the overview picture and MNs was put on the center of the base. The top was a pressure sensor which would move downward to press the MNs and record the value at the same time. Thus, mechanical performance of MNs with different exposure time could be obtained by the testing machine. 

**Figure 3:**
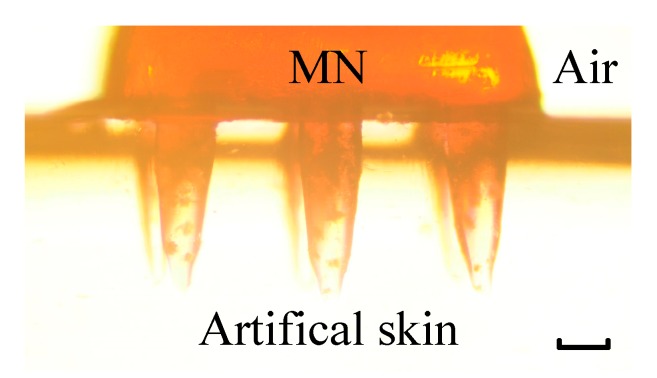
The state MNs was stuck into artificial skin (5 wt% alginate hydrogel). The scale bar is 200 µm.

After mechanical tests of MNs, the balance of between precision and stiffness was found. Then to certify the skin penetration, drug injection, and detection ability of MNs, 5 wt% alginate hydrogel was used as artificial skin in drug injection and drug detection experiments. Since 5 wt% alginate hydrogel has similar mechanical properties to human skin [24,25]. Alginate hydrogel was prepared by following method: Take 1 g alginate (Aladdin) into a glass beaker, add 20 mL DI water, and stir for 10 min. Then add 0.5 g Gluconolactone (Aladdin) and 0.5 g Disodium calcium (Aladdin) to solution and stir for 5 min. Pour solution into plastic dish. Then hydrogel would solidify within hours. The skin penetration simulation was similar to the method in Figure 2, but MNs was adhered to the surface of the sensor. As shown in Figure 3, needles of MNs were stuck into alginate hydrogel, but the base of MNs was still exposed to air. In drug injection experiment, MNs were soaked in Rhodamine B solution for 24 h. Then MNs were stuck into normal alginate hydrogel. By capturing the pictures in fluorescence microscope, the relative concentration of rhodamine B was known. As for drug detection experiment, only alginate hydrogel was soaked in rhodamine B solution for 24 h.

## 3. Results

### 3.1. Influence of Exposure Time

**Figure 4:**
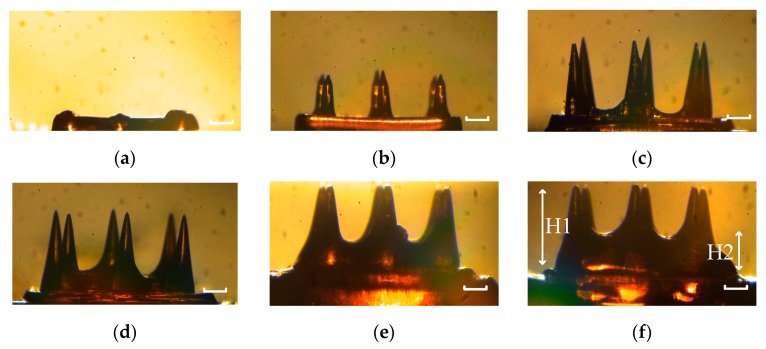
The picture of side face of MNs with different exposure time: (**a**) 50 ms; (**b**) 100 ms; (**c**) 300 ms; (**d**) 500 ms; (**e**) 700 ms; and (**f**) 900 ms. The scale bar is 200 µm.

Exposure time has great influence on the mechanical performance of printed MNs. Long exposure time could enhance the stiffness of MNs, however, MNs were bigger than designed MNs. Short exposure time could avoid over exposure, but the stiffness of MNs would decrease. It is crucial to find a balance between precision and stiffness. In this section, the impact of exposure time on precision was analyzed in detail. MNs with exposure time ranging from 50 ms to 900 ms were printed, and the side face pictures of those MNs are shown in Figure 4. The needles of MNs of which the exposure time was 50 ms could not be printed, because the light density may be under the threshold. The needles of MNs of which the exposure time was 100 ms could not be totally printed, since the light density may decrease under the threshold when sliced images came close to the tip. As for MNs with 300 ms exposure time, the needles were built well, and they had no sign of over exposed. For MNs with 500 ms, 700 ms, and 900 ms exposure time, all of them were over exposed in different degree. Besides, the interval between needles were filled higher as exposure time went up.

The real height of needles was defined as H1, and the curing height of the interval between needles was defined as H2. H1 and H2 were displayed in Figure 4e. And the height of needle designed in model (H0) was 700 µm. Moreover, H1/H2 was used to qualify the impact of over exposure. All those data are displayed in Table 1 in details.

### 3.2. Mechanical Test of Microneedles

#### 3.2.1. The Stiffness of Microneedle

**Figure 5:**
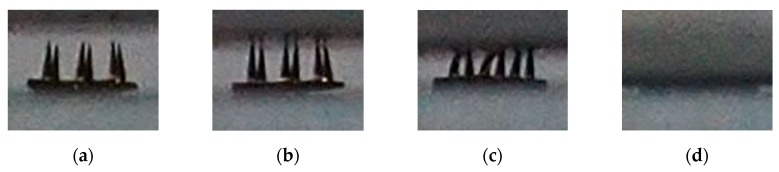
The side face picture of pressing process: (**a**) before contraction; (**b**) tip contact the sensor; (**c**) the needle deformed under the pressure; and (**d**) the MNs was crushed totally.

The side face pictures of mechanical properties testing process are displayed in Figure 5. At the very beginning, MNs was free of pressure. As sensor went downward and contact with MNs, MNs started to deform and push against the sensor. Finally, MNs was totally crushed. The contracting process stopped until detected pressure achieved pre-set value. The overview environment is displayed in Figure 2.

**Figure 6:**
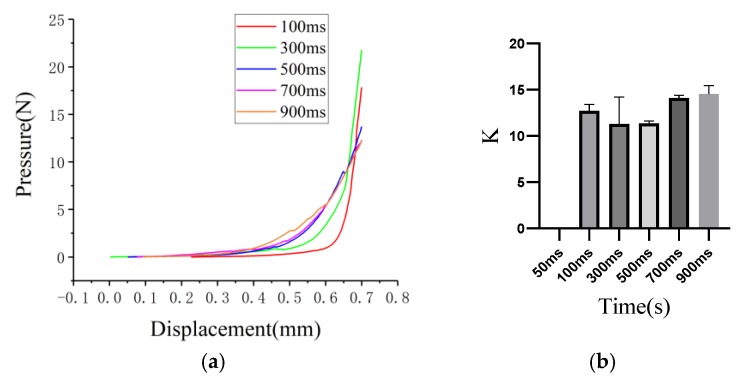
The mechanical properties of MNs with different exposure time: (**a**) the pressure-displacement curves of MNs with different exposure time; (**b**) the K of MNs with different exposure time.

The impact of exposure time on the mechanical properties of MNs is shown in Figure 6a. Due to the short time exposed to blue light, MNs with 100 ms exposure time had significantly shorter needle than others in experiments. While MNs with 100 ms exposure time were shorter, a fixed coordinate system was used in pressure-displacement experiments. And as a result, the pressure in almost all curves started to increase at approximate displacement. The decay happened in blue curve were caused by the fracture of needles during contracting process. When MNs was totally crushed, the slope of the curve was equal to young’s module of steel base.

K was defined as

K = (P_2_ − P_1_)/(D_2_ − D_1_), (1)

where P_1_,D_1_ were coordinate values of starting point in curves and P_2_,D_2_ were coordinated values of changing point next to starting point. It represented the pressure increased after the displacement increased and could indicate the stiffness of MNs. As exposure time increased, the K of MNs ascended at the same time according to Figure 6b. MNs with 50 ms exposure time could not be built so that the K of 50 ms was 0. Since MNs with 100 ms were not pressed from the designed height of the needle due to shorter needle length than MNs with longer exposure time, so the results were reasonable.

Comparing to other MNs, MNs with 300 ms exposure time have well precision and stiffness. In consequence, 300 ms MNs were used in following drug injection and drug detection experiments. The influence of water content is shown in Figure S1.

#### 3.2.2. Penetration of Artificial Skin

**Figure 7:**
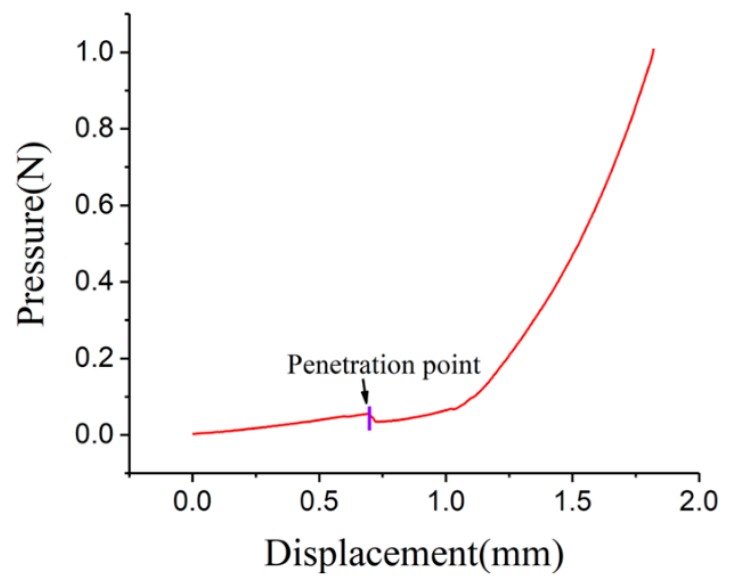
The pressure-displacement curve of artificial skin penetration.

Penetration test was also performed by the universal material testing machine while the MNs and artificial skin were fixed on the sensor and the base, respectively. The pressure-displacement curve of artificial skin penetration process is shown in Figure 7. During the simulation, needle and artificial skin would deform once they contact with each other. Thus, there was a rise on pressure as displacement increased. As pressure increased, artificial skin would not bear the pressure and was plunge by MNs finally. Decline happened on pressure was signed with purple bar and the decline point was penetration point. As a consequence, the MNs were enough tough and had the ability to penetrate artificial skin.

### 3.3. Drug Delivery Performance of Microneedle

**Figure 8:**
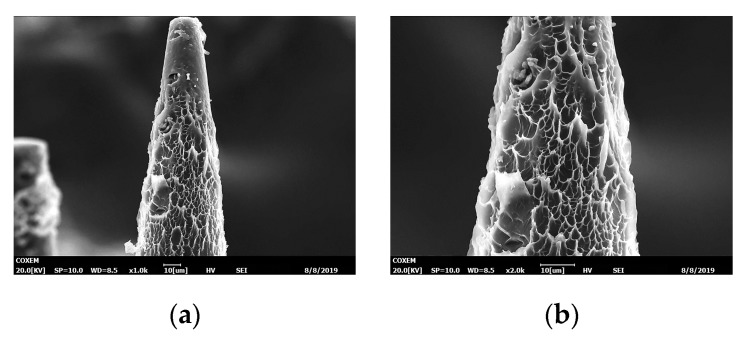
SEM of MNs with 300 ms exposure time: (**a**) tip of a needle; (**b**) surface of tip area. The scale bar is around 10 µm.

MNs were fabricated from precursor with the help of blue light. Monomers crosslinked with each other and formed grid structure. Thus, MNs had many micro pores on its surface as shown in Figure 8. The scale bar of Figure 8 is around 10 µm. These pores could increase the contacting area significantly and may act as transportation channel for material exchange. Therefore, it was a quick process for drug to enter into hydrogel or tissue from MNs when MNs were stuck into them. 

#### 3.3.1. Simulation of Drug Injection

**Figure 9:**
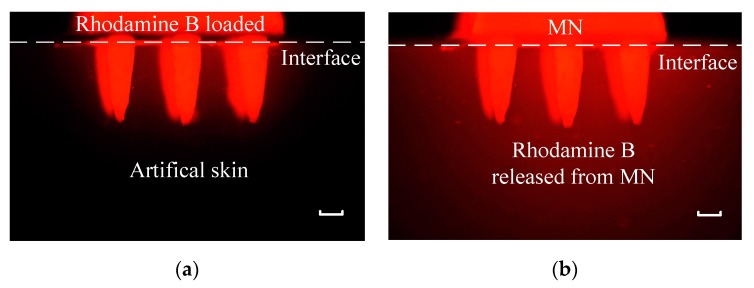
Fluorescence pictures of MNs with rhodamine B stuck into artificial skin without rhodamine B: (**a**) the moment MNs was stuck into artificial skin; (**b**) the MNs had been stuck into artificial skin for 1 h. The scale bar is 200 µm.

As described in Figure 3, during simulation of drug injection, MNs was soaked in 0.005 wt% rhodamine B solution for 24 h while artificial skin was soaked in DI water for 24 h. Then MNs with rhodamine B was stuck into artificial skin which did not contain rhodamine B at first. As rhodamine B could release red fluorescence at 580 nm when exposed to green laser at 532 nm, samples were put under 532 nm wavelength green laser and fluorescence picture was taken every 2 min. The initial state and final state pictures were displayed in Figure 9a,b, respectively. Brightness and contrast ratios of two pictures were enhanced via software to get a better exhibition. Top was MNs with rhodamine B while bottom was artificial skin without rhodamine B at first. The dotted white line represented the interface of MNs and artificial skin. The artificial skin was black under green laser and became red after 1 h when it was under green laser again. 

**Figure 10:**
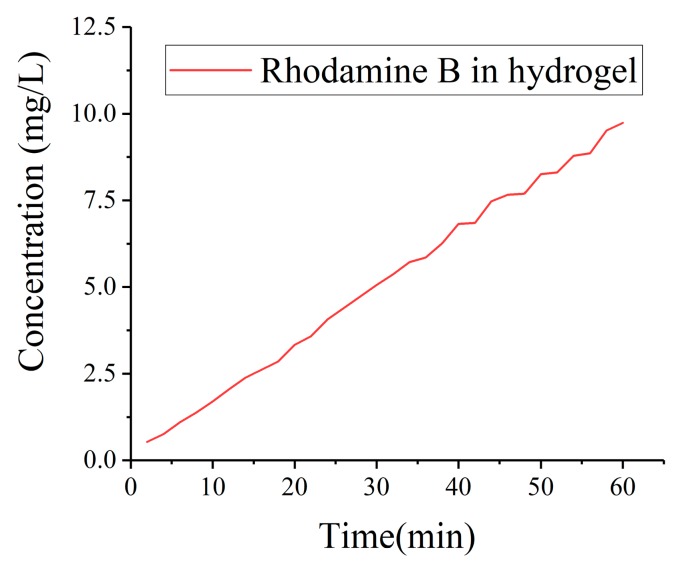
Integrated Density of rhodamine B in artificial skin.

The images of artificial skin area in all recorded pictures were imported into Matlab and transferred into gray pictures. The gray values were calculated as followed: Summing gray values of every pixel up, then the total value was divided by the numbers of pixels. Mean integrated density was obtained by methods above. The concentration is calculated as shown in Figure S2. And the quantitative data of drug injection is displayed in Figure 10. It was obviously that the concentration increased significantly as time increased. Time spent on plugging MNs into artificial skin was necessary so that the initial recording time was 2 min. Since rhodamine B had already penetrated into artificial skin by 2 min, the integrated density was above zero. The integrated density went up as time increased, which indicated that MNs have well drug injection ability.

#### 3.3.2. Drug Detection

**Figure 11:**
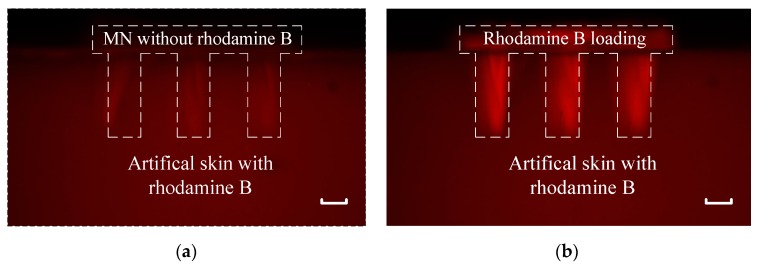
Fluorescence pictures of MNs without rhodamine B stuck into artificial skin with rhodamine B: (**a**) the moment MNs was stuck into artificial skin; (**b**) the MNs had been stuck into artificial skin for 30 min. The scale bar is 200 µm.

Similar method in drug injection was used in simulation of drug detection. MNs was soaked in DI water for 24 h while artificial skin was soaked in 0.005 wt% rhodamine B solution for 24 h. Then MNs without rhodamine B was stuck into artificial skin with rhodamine B. Samples were put under green laser at 532 nm and fluorescence picture was taken every 2 min. The initial state and final state pictures are displayed in Figure 11a,b, respectively. Brightness of two pictures were reduced while contrast ratios were also enhanced by software. Top was MNs without rhodamine B while bottom was artificial skin with rhodamine B. The dotted line in pictures were the outline of MNs under 532 nm wavelength green laser. The needle of MNs was not bright at the very beginning. As rhodamine B enter into the needle, the fluorescence in needle became bright. 

**Figure 12:**
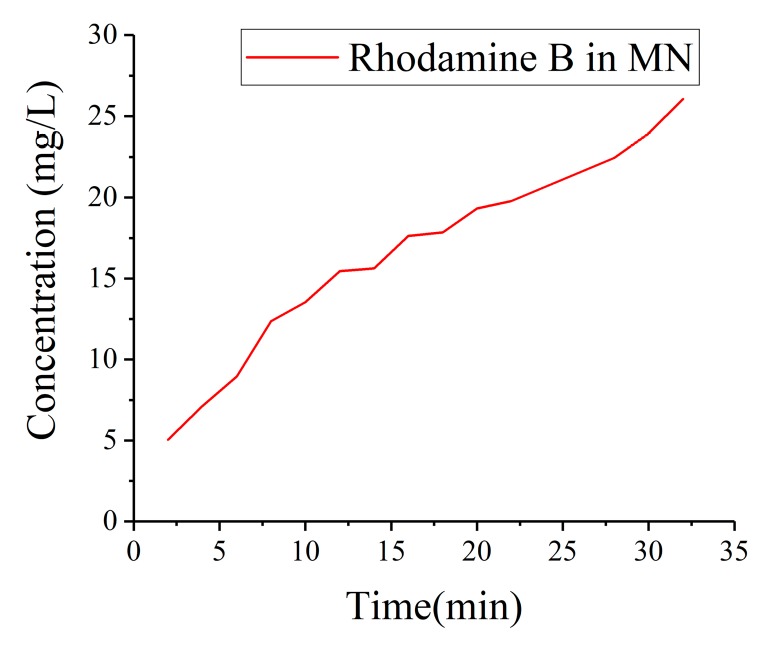
Integrated Density of rhodamine B in white rectangle area in MNs.

The images of white dotted area in all pictures recorded were imported into Matlab and transferred into gray pictures. The same method was used to calculated the mean integrated density. The quantitative data of drug detection is displayed in Figure 12. It was obviously that the concentration increased significantly as time increased. Time spent on plunge MNs into artificial skin was necessary so that the initial times was 2 min. However, the penetration was much swifter than we thought. At 2 min, the base which was black without rhodamine B at first had already became a little red. The integrated density was over 20 at 2 min from Figure 12.

## 4. Discussion and Conclusions

3D building is a novel and low-cost method in manufacturing MNs comparing to conventional template driven fabrication. We could use H-P DLP system to build MNs into many shapes needed with bio-compatible materials in different printing parameters. The precision and stiffness of MNs could be controlled by the exposure time. When exposure time as over 300 ms, the precision would decline as exposure time ascend while the stiffness would increase at the same time. There was a balance between precision and stiffness. After groups of mechanical tests and measurement, we regard 300 ms as an ideal exposure time for building MNs. 

There are limitations of existed MNs such as solid MNs, coated MNs, and commercial 3D printed MNs. Solid MNs is relatively rough and could cause irreversible wound and dosage of coated MNs is limited, besides, it is difficult to control and quantify [10,11]. Photosensitive resin used in SLA has poor biocompatibility [19,20]. Combining template driven fabrication with UV curing materials is a complicated process [21,22]. Comparing with those microneedles, hydrogel microneedles could be used for drug loading and the drug loading capacity is greatly increased. The fabrication time of biocompatible microneedles is greatly reduced owe to the ordinal fabrication of base and needles. The fabrication of several microneedles could be completed only in few minutes.

In this paper, we have done the experiments and result analysis of printing parameters on the mechanical properties of microneedles, which provides a method to change the mechanical properties of microneedles. In addition, we also achieved the simulation experiments of micro needle puncture of artificial skin and the simulation experiments of drug injection and drug extraction by MNs. What is more, the results of drug injection and drug detection were analyzed quantitatively, which may provide a certain reference for micro needle using in biological injection and drug extraction. Due to the low-cost, painless, short manufacturing time, well biocompatibility, and drug delivery performance properties of 3D built MNs, it is exciting to adapt MNs to the market. We expect to apply 3D printed MNs with properties above to clinical usage in the future.

## Figures and Tables

**Table 1:** Parameters of MNs

Exposure Time (ms)	H1 (mm)	H2 (mm)	H2/H1 (%)	H0 (mm)
50	0.044	0.000	0.0	0.700
100	0.451	0.000	0.0	0.700
300	0.732	0.043	5.9	0.700
500	0.748	0.113	15.1	0.700
700	0.781	0.251	32.1	0.700
900	0.812	0.349	43.0	0.700

## Supplementary Materials

The following are available online at https://www.mdpi.com/2072-666X/11/1/17/s1, Figure S1. The impact of hydration on the mechanical properties and scale of microneedles (MNs): (a) The mechanical properties of MNs with different water content; (b) The bottom width of needle with different water content, Figure S2. The integrated density corresponding to different concentration of rhodamine B, Video S1: Mechanical performance testing.
