# Supplementary material for: 3D Printed Multi-Functional Hydrogel Microneedles Based on High-Precision Digital Light Processing
Source: Micromachines (Basel). 2019 Dec 23;11(1):17. doi: 10.3390/mi11010017 (PMC7019295; doi:10.3390/mi11010017)
Supplement: Supplementary file 1 [file micromachines-11-00017-s001.zip › micromachines-618631-suppl/micromachines-618631-supplementary.docx]

Supplementary Information: 3D Printed Multi-Functional Hydrogel Microneedles Based on High-Precision Digital Light Processing

Wei Yao ^1^, Didi Li ^1^, Yuliang Zhao ^2^, Zhikun Zhan ^3^, Guoqing Jin ^4^, Haiyi Liang ^5,6^
and Runhuai Yang ^1,^*

^1^ Department of Biomedical Engineering, Anhui Medical University and Research and Engineering Center of Biomedical Materials, Anhui Medical University, Hefei 230032, China; [yaowei971020@163.com](javascript:void(0);) (W.Y.); [lidi970113@gmail.com](mailto:lidi970113@gmail.com) (D.L.)

^2^ School of Control Engineering, Northeastern University at Qinhuangdao, Qinhuangdao 066004, China; zhaoyuliang@neuq.edu.cn

^3^ Key Lab of Industrial Computer Control Engineering of Hebei Province, School of Electrical Engineering,Yanshan University, Qinhuangdao 066004, China; zkzhan@ysu.edu.cn

^4^ Robotics and Microsystems Center, School of Mechanical and Electric Engineering, Soochow University, Suzhou 215021, China; gqjin@suda.edu.cn

^5^ CAS Key Laboratory of Mechanical Behavior and Design of Materials, University of Science and Technology of China, Hefei 230027, China; hyliang@ustc.edu.cn

^6^ IAT-Chungu Joint Laboratory for Additive Manufacturing, Anhui Chungu 3D printing Institute of Intelligent Equipment and Industrial Technology, Wuhu, Anhui 241200, China

***** Correspondence: yangrunhuai@ahmu.edu.cn; Tel.: +86-551-6516-1265

Water content of microneedle (MN) would change with time and it have impacts on the dimensions and mechanical properties of MNs. While the water content of hydrogel decreased, the stiffness of MN was enhanced. However, hydrogel would shrink slightly when water content decreased. Groups of experiments were performed to qualify the hypothesis. MNs were exposed to air of which the humidity was 19 % for different hours before mechanical tests to control the water content. The water content of MNs would vary with exposure time and that longer exposure time caused lower water content. The results of mechanical tests are shown Supplementary Figure S1a, the pressure curve of MN exposed to air for 12 h increased most significant as displacement increased. MNs would break during the testing process, there are damping in curves as consequence. MNs were also soaked into DI water for same hours as those exposed to air. Benefitting from the high swollen ratio of hydrogel, MNs had well drug loading ability. Although the scale of MNs changed significantly after soaking. Thus, the curves of mechanical properties were irregular. The scale of MNs exposed to air or soaked in DI water is also shown in Supplementary Figure S1b. MNs after soaking in drug could be placed under environment with certain humidity before functioning.

| **** | **** |
| --- | --- |
| (**a**) | (**b**) |

**Figure S1.** The impact of hydration on the mechanical properties and scale of microneedles (MNs): (**a**) The mechanical properties of MNs with different water content; (**b**) The bottom width of needle with different water content.

**Figure S2.** The integrated density corresponding to different concentration of rhodamine B.

To quantify rhodamine B released or loaded, from or into MNs in previous experiments, fluorescence pictures of hydrogels soaked in rhodamine B solutions for 24 h were taken. Rhodamine B solutions had a concentration gradient of 10 mg/L ranging from 0 mg/L to 50 mg/L. Fluorescence pictures recorded were imported into Matlab and transferred into gray pictures. The gray values of every pixel were summed up, and the total value was divided by the number of pixels. Then the average integrated density was obtained. The standard curve of integrated density and concentration was fitted and shown in Supplementary Figure S2.

Due to the high concentration of rhodamine B solution, the fitting curve does not represent linearity totally. The result of drug detection after 30 min was marked with blue line and the result of drug injection after 1 h was marked with green line. Figures 10 and 12 were replaced by concentration-time curve.
